# Building Water Models Compatible with Charge Scaling Molecular Dynamics

**DOI:** 10.1021/acs.jpclett.4c00344

**Published:** 2024-03-07

**Authors:** Victor Cruces Chamorro, Pavel Jungwirth, Hector Martinez-Seara

**Affiliations:** Institute of Organic Chemistry and Biochemistry, Czech Academy of Sciences, Flemingovo nám. 2, 16610 Prague 6, Czech Republic

## Abstract

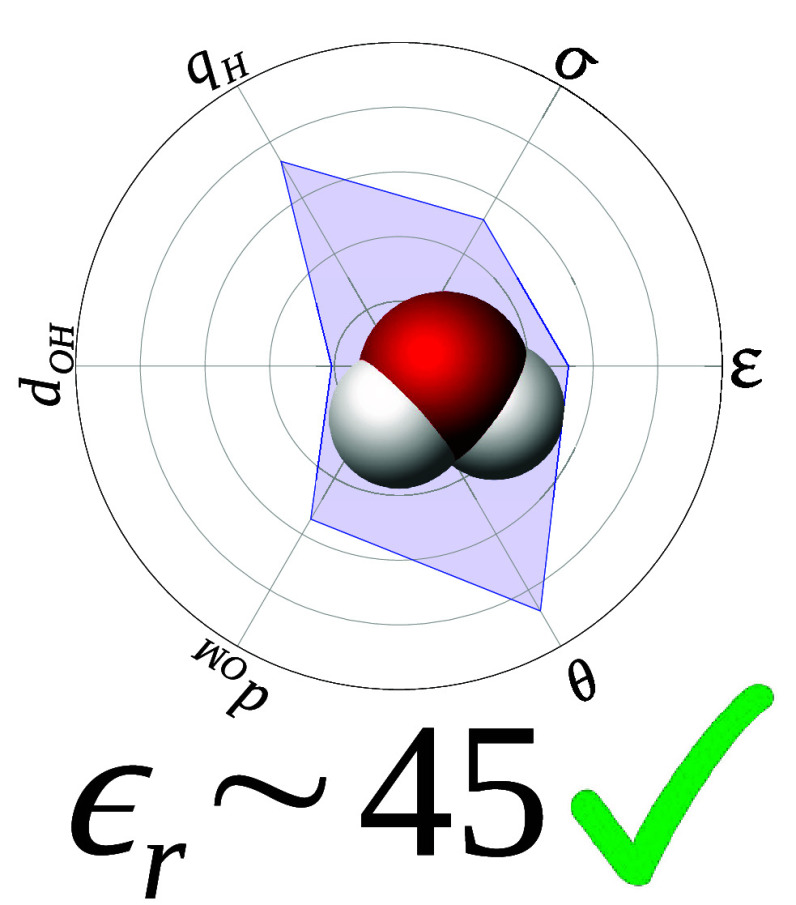

Charge scaling has proven to be an efficient way to account in a mean-field manner for electronic polarization by aqueous ions in force field molecular dynamics simulations. However, commonly used water models with dielectric constants over 50 are not consistent with this approach leading to “overscaling”, i.e., generally too weak ion–ion interactions. Here, we build water models fully compatible with charge scaling, i.e., having the correct low-frequency dielectric constant of about 45. To this end, we employ advanced optimization and machine learning schemes in order to explore the vast parameter space of four-site water models efficiently. As an a priori unwarranted positive result, we find a sizable range of force field parameters that satisfy the above dielectric constant constraint providing at the same time accuracy with respect to experimental data comparable with the best existing four-site water models such as TIP4P/2005, TIP4P-FB, or OPC. The present results thus open the way to the development of a consistent charge scaling force field for modeling ions in aqueous solutions.

Water molecules are ubiquitous in living systems and technological applications due to their physicochemical properties that make water a unique universal solvent.^1^ Water thus provides an environment where life and chemistry take place by dissolving molecules and ions, allowing specific molecular and supramolecular structures, and directly contributing to stabilizing interactions and catalyzing reactions. Force field molecular dynamics simulations (FFMD) represent a powerful tool for modeling these biological and technological processes with atomistic resolution at femtosecond to millisecond time scales. The first simulations involving water date back to the early days of FFMD.^2^ Consequently, the development of empirical potentials for water has been a recurrent topic in the past decades (e.g, TIPS,^3^ SPC,^4^ TIP3P,^5^ SPC/E,^6^ and TIP4P^7^ models) and is far from settled^8^ (e.g., the more recent TIP4P/2005,^9^ TIP4P-FB,^10^ and four-site OPC^11^ (OPC4) models). Aqueous solutions have proven to be difficult systems to describe accurately and are thus an active area of research.^12^ Even pure water behavior is not easy to model such that it accurately covers the full range of biologically relevant thermodynamic conditions.^8^

Commonly used water potentials were typically optimized to recover selected experimental or calculated data. Therefore, they reproduce these target properties at the optimization conditions, but there is no guarantee that they will also reproduce other properties or the target properties at different thermodynamic conditions. The optimization process traditionally focuses on properties derived from the density^13^ and the self-diffusion coefficient,^14^ while other properties, such as the surface tension or the dielectric constant,^15^ are given a secondary role or not optimized at all. It is thus not surprising that their values vary significantly between existing models^11,16^ despite their physical relevance.^17^

In particular, the dielectric constant (ε_*r*_) is an essential property dictating how interactions between charged particles are attenuated in a given medium. The dielectric constant can be approximately split into two contributions of different origins.^18^
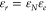
1

The nuclear contribution to the dielectric constant (ε_*N*_) accounts for the “slow” rearrangement of atomic nuclei of water molecules as a response to changes in local or external electromagnetic fields. In contrast, the electronic contribution to the dielectric constant (ε_*e*_) accounts for the “instantaneous” response of the electronic clouds of the water molecules and can be approximated by the square of the refraction index^19^ (ε_*e*_ ≈ *n*^2^ = 1.78).^20^

FFMD that lacks polarization terms accounts only for the nuclei contribution of the dielectric response of the medium. One could potentially employ the computationally more demanding polarizable force fields such as Drude^21^ or Amoeba^22^ to capture the electronic contribution of the response. As an alternative, one can introduce the missing electronic polarization in a mean-field way denoted as the electronic continuum correction (ECC).^20,23,24^ Within this approach, the system is immersed in an electronic dielectric continuum, which is mathematically equivalent to scaling the ionic charges by the inverse square root of the electronic part of the dielectric constant of the medium .

The ECC framework circumvents the problem of explicitly accounting for electronic polarization for interactions between dissolved ions or charged groups. There is, however, a catch—existing nonpolarizable water models often exhibit values of dielectric constants larger than ε_*N*_, effectively transferring (part of) the missing ε_*e*_ to ε_*N*_. They also possess water dipole moments larger than the gas phase value (albeit typically smaller than the value in the liquid).^25^ Employing currently available water models thus results in an artificial overscaling when used within the ECC approach.^23^

Within this study, we succeeded in developing a class of four-site water models compatible with the ECC approach (i.,e, possessing ε_*r*_ ≈ 45), which are comparable in predicting experimental observables to the best of the existing four-site water models (possessing significantly larger values of ε_*r*_). Considering the above constraint of a low dielectric constant, it was not clear from the onset whether such a model can be developed.

Our target four-site water models are fully defined by six parameters, see Table 1. Similarly, as in the TIP4P family of models, these are the Lennard-Jones parameters (i.e., σ and ε) on the oxygen atom (with no explicit van der Waals terms on the hydrogens), the charge on each of the hydrogen atoms (*q*_*H*_) (that also defines the charge on the dummy atom *q*_*M*_ = −2*q*_*H*_), and the intramolecular parameters. Namely, these are the oxygen–hydrogen (*d*_*OH*_) and oxygen–dummy atom (*d*_*OM*_) distances and the hydrogen–oxygen–hydrogen angle (θ). Note that the dummy atom is placed at the bisector of the angle θ in the direction toward the hydrogen atoms.

**Table 1:** Optimized Parameters with Boundaries and Seeding Values

Parameter	Units	Boundaries	Initial
σ	nm	0.3050–0.3250	0.3150
ε	kJ/mol	0.5000–1.0000	0.7500
*q*_*H*_	e^–^	0.3500–0.7000	0.5500
*d*_*OH*_	nm	0.0900–0.1000	0.0960
*d*_*OM*_	nm	0.0120–0.0180	0.0150
θ	deg	100.00–110.00	105.00

On the technical side, developing an empirical force field is a computationally expensive and time-consuming endeavor, primarily due to the large number of simulations required for testing extensive sets of parameters. For us to effectively tackle water force field development, we need a framework that reduces the number of simulations ultimately performed while still being able to localize the optimal regions of the parameter space. To this end, we have developed an automated framework that efficiently avoids sampling suboptimal regions of parameter space using a combination of artificial intelligence (AI) tools and other advanced optimization methods (Figure 1).

**Figure 1:**
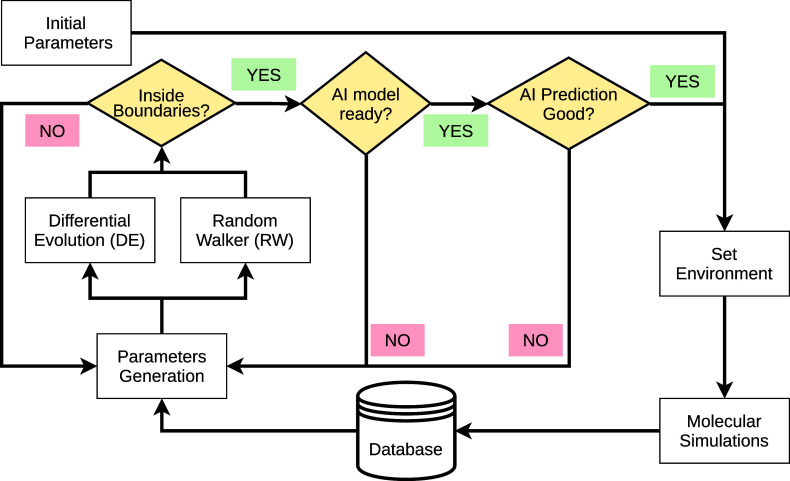
Scheme of the program routine used to generate new parameters.

To avoid any bias and to critically evaluate our developed framework for sampling the parameter space, we do not explicitly assume any concrete relationship between parameters and target properties when starting the optimization process (although such constraints could be easily incorporated). Under such conditions, random walkers (RW) are useful for an initial sampling of the parameter space and for gathering information about their relationship with target physical properties. Additionally, RW improves simulation stability because it uses the last molecular configuration of the previous point as a starting configuration for the new simulation point. This is an important feature when using an automatic framework. RW simulations started from the parameters presented in Table 1. In addition, boundaries were set to keep the water geometry and physical properties within reasonable limits, see Table 1. The resulting parameter space is large enough to encompass both good as well as less optimal regions without enforcing initial biases while simultaneously avoiding sampling of physically unreasonable regions. In particular, to sample the present six-dimensional space, we performed using RW 1000 simulations at 300 K and 1 bar, exploring a relatively wide ε_*r*_ range.

Once the parameter space is sparsely sampled by the above approach, a second phase begins where we optimize the process of generation of parameters using the differential evolution (DE) algorithm.^26^ This algorithm generates new parameter sets or points as a linear combination of the parameters from the best points of the available population, i.e., the previously obtained points. Such a method efficiently parallelizes the optimization process while simultaneously improving the sampling capacity, which is crucial when dealing with high-dimensional problems such as force field development. The price to pay is that such parameter generation, being stochastic in nature, does not ensure that the new candidate is necessarily better than the existing points. Only when the generated candidate improves the quality of the parameters in terms of the accuracy of the simulated target properties is it used in subsequent steps by DE. The optimization process is finished once the population of parameters has reached the desired convergence. In this work, this corresponds roughly to 4500 sets of parameters. Considering that one needs to ultimately test the generated parameters by performing FFMD and that DE may generate (particularly at the beginning of the optimization process) points which are far from optimal regions, there is a need for further streamlining the whole process.

The parameter convergence can be significantly accelerated, i.e., the number of simulations needed to be performed can be reduced if we introduce a method that estimates the output results for the DE suggested parameter sets or points without actually running the simulations. As shown in Figure 1, we can use a mapper function to predict the outcome of the candidate such that if the predicted outcome is worse than a predefined value of the target cost function, the program skips the actual simulation and directly generates a new candidate. The mapper function used in this work is a fully connected multilayer neural network. The input layer vector contains all our parameters normalized from 0 to 1. The ReLu activation function is used in the four hidden layers connected by a dropout layer with a rate of 0.10, each layer having 40 nodes which cannot have a bigger norm than 5.0. A linear activation function is used for the output layer. Finally, the neural network is trained using early stopping such that we avoid possible overfitting while conserving the prediction capacity of the neural network.^27^

In this work, we build the neural networks used as mapping functions employing the data obtained from all simulations performed so far. As creating neural networks is very fast compared to performing simulations, they are recreated whenever new data is available, i.e., when new simulations are performed. While initially the neural network’s performance is not yet optimal, even at this point, it is often sufficient to discriminate bad points. Also, the fact that good points are occasionally wrongly rejected does not affect the convergence significantly since these can be sampled at a later time as the prediction capability of the neural network improves upon being trained with an increasing amount of data. More data also reduces overfitting, which would otherwise negatively impact the prediction capabilities of the neural network. Using the finally obtained well-performing neural network, an efficient refinement algorithm described in the Supporting Information was used to increase the sampling capacity further, improving the obtained water models. A point is considered better than a previous one when it lowers a cost function that expresses the weighted difference between reference and simulation values for our selected target experimental properties, see Table 2. Note that for the diffusion constant *D*_*OW*_, we have scaled the experimental value^14^ used for comparison to adjust to the effect of the finite size of the simulated unit cell.^28^ For optimization of the dielectric constant, the cost function (*C*_*ECC*_) considers as ε_*r*_ only the nuclear contribution to the experimental dielectric constant to be compatible with the ECC approach.^23^ Otherwise, ε_*r*_ is not included in the cost function (*C*_*G*_). Our cost function reads as
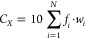
2where *f*_*i*_ and *w*_*i*_ are the loss function and weight for each property, respectively. Here, we use mean absolute percentage error (MAPE), normalized to 1, as a loss function to calculate the deviation between a given simulation property and experiments. Being a percentage-based metric, it is scale-independent, making it useful for comparing the accuracy of properties on different scales. The cost function is a weighted average, see eq 2, where the weights are normalized to sum to 1.

**Table 2:** Reference Properties and Functional Parameters Used in the Optimization Process^a^

	Values	Units	Loss function	Weight^b^
ρ_1bar_	Table S2	kg/m^3^	MAPE	0.667
ε_*r*_	44.5	—	MAPE	0.111
*D*_*OW*_	2.16 × 10^–5^	cm^2^/s	MAPE	0.111
rdf_1*p*_	0.280	nm	MAPE	0.0555
rdf_1*h*_	2.58	—	MAPE	0.0555

a ρ_1bar_ are density values at 1 bar from 260 to 360 K every 20 K. ε_*r*_ is the relative permittivity according to the ECC approach.^23^*D*_*OW*_ is the experimental self-diffusion coefficient of water at 300 K and 1 bar accounting for our simulation of 832 water molecules using Hummer-Yeh periodic boundary conditions correction.^28^ rdf_1*p*_ and rdf_1*h*_ are the position and height of the first oxygen–oxygen RDF peak. The weights of the properties, ensuring a balanced sampling of all the properties, are normalized to sum to 1.

b The weights correspond to *C*_*ECC*_.

All FFMD simulations for the optimization process were performed using the GROMACS2019 molecular dynamics package.^29^ The number of water molecules in the cubic simulation box is 832. This number was chosen because it is small enough for an efficient optimization process but large enough (i.e., minimum unit cell size of 2.70 nm) to fulfill the minimum image convention and the corresponding cutoffs. Namely, we employed an interaction cutoff of 1.2 nm for the particle mesh Ewald (PME)^30^ and the PME Lennard-Jones schemes that take into account the long-range electrostatic and van der Waals interactions. We used the leapfrog algorithm with a time step of 2.0 fs and a total simulation time of 21 ns. The first nanosecond was considered equilibration and skipped for the analysis. The isothermic-isobaric (NpT) ensemble was enforced using the Nosé–Hoover thermostat^31^ with a relaxation time of 1.0 ps and the Parrinello–Rahman barostat^32^ with a compressibility of 5 × 10^–5^ bar^–1^ and a relaxation time of 5.0 ps.

The results of the optimization process are summarized in Figure 2. A total of 1343 parameter sets were generated within the optimization process possessing ε_*r*_ values between 40 and 50, i.e., very close to the value of 45 fully compatible with the ECC approach. From these, there is a sizable region in the parameter space with an acceptably small deviation from experiments (*C*_*ECC*_ < 0.7). This region includes 791 points. For comparison, a widely used three-site model TIP3P possesses a much larger value of *C*_*G*_ = 1.973. To further illustrate the performance of these points, we categorize them in two additionally constrained regions with *C*_*ECC*_ < 0.5 and *C*_*ECC*_ < 0.3. As discussed below, the latter corresponds to models with performance comparable to that of current state-of-the-art four-site water force fields. The optimal ECC water force field region with *C*_*ECC*_ < 0.3 occupies a well-defined region of parameters σ ≈ [0.315–0.316] nm, ε ≈ [0.65–0.825] kJ/mol, *q*_*H*_ ≈ [0.51–0.64] *e*^–^, *d*_*OH*_ ≈ [0.90–1.0] nm, *d*_*OM*_ ≈ [0.135–0.180] nm, and θ ≈ [106–110]°. Also, note that the 50 best-performing models are spread fairly evenly in this optimal region. This suggests a rather flat cost-optimal region in the parameter space compatible with ECC. An extended view of the sampled parameter space as a function of the resulting cost function is presented in Figure S2.

**Figure 2:**
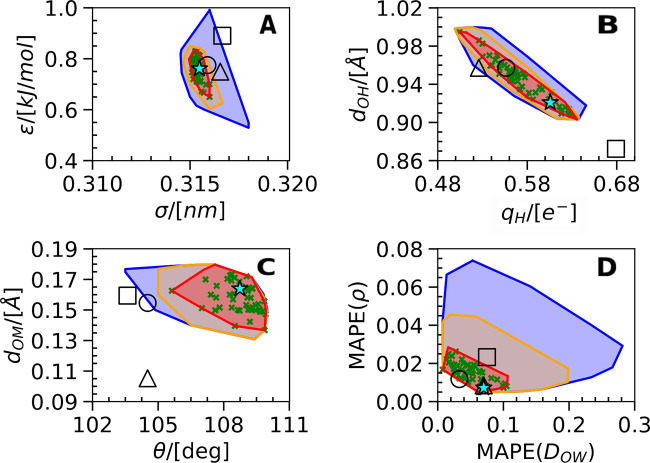
Minimum convex polygon (convex hull) that contains all points inside a region for selected pairs of parameters or properties: (A) σ and ε, (B) *q*_*H*_ and *d*_*OH*_, (C) θ and *d*_*OM*_, and (D) mean percentage errors of *D*_*OW*_ and ρ. The regions are defined by the scoring values points: blue (*C*_*ECC*_ < 0.7), orange (*C*_*ECC*_ < 0.5), and red (*C*_*ECC*_ < 0.3). The green symbols (×) are our best 50 points with ECCw2024 denoted as (★). The black open symbols correspond to TIP4P/2005 (○), OPC4 (□), and TIP4P-FB (△).

To contextualize our optimal region, we compare its performance to that of existing state-of-the-art four-site water models simulated under the same conditions (the empty black symbols in Figure 2 correspond to TIP4P/2005 (○), OPC4 (□), and TIP4P-FB (△)). Two of these water models (i.e., TIP4P/2005 and TIP4P-FB) are of a fixed gas phase geometry, while OPC4 and our models optimize the water geometry parameters (see Table 3 for a complete list of their parameters). Note that σ and ε values and the charges *q*_*H*_ of all these water models fall within a narrow region for *C*_*ECC*_ < 0.7, which seems to be highly preserved (especially for σ) for water models.^16^ The bond parameters *d*_*OH*_ and *d*_*OM*_ of these models also fall within the optimal region with the exception of *d*_*OM*_ for TIP4P-FB that is 30% smaller. Finally, the θ parameters of these models are at the edge of our optimal region. In summary, our results demonstrate that despite the constraint of the target ε_*r*_ compatible with ECC, the optimal parameter region is sizable and robustly defined.

**Table 3:** Parameters of the Water Models Used in This Publication^a^

Param.	ECCw2024	TIP4P/2005	TIP4P-FB	OPC4
*d*_*OH*_[nm]	0.092084	0.09572	0.09572	0.08724
θ[deg]	108.7392	104.52	104.52	103.60
σ[nm]	0.315480	0.31589	0.31655	0.316655
ε[kJ/mol]	0.761154	0.7749	0.74928	0.89036
*q_H_*[au]	0.605689	0.5564	0.52587	0.6791
*d*_*OM*_[nm]	0.016388	0.01546	0.010527	0.01594
μ[*D*]	2.167631	2.305097	2.427804	2.479542
*Q*_*T*_[*D*Å]	2.444435	2.296802	2.170775	2.299607

a TIP4P/2005 and TIP4P-FB have the gas phase molecular geometries. OPC4 and ECCw2024 allow different molecular geometries during their optimization.

Among all the ECC-compatible models in the optimal region (*C*_*ECC*_ < 0.3), we present here in detail one of the best performing models in terms of the cost function (*C*_*ECC*_ = 0.231/*C*_*G*_ = 0.262) while possessing a balanced structural, thermodynamic, and dynamic behavior, see Table 4. The quality of our model, which we label as ECCw2024, is comparable to that of existing four-site models such as TIP4P/2005, OPC4, or TIP4P-FB, see Table 4 and Figure 3. This is a nontrivial result, allowing further force field development with a water model fully compatible with the ECC framework, i.e., possessing a dielectric constant of about 45. Table S2 contains the numerical values for each of the evaluated properties for these models.

**Figure 3:**
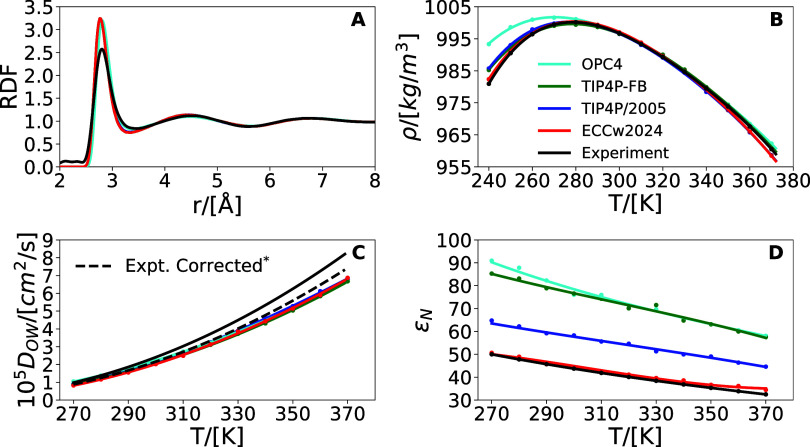
Water model performance in comparison with water experimental results. (A) The radial distribution function^33^ at 300 K and 1 bar. (B) Density isobar at 1 bar.^13^ (C) *D*_*OW*_ isobar at 1 bar.^14^ (D) ε_*N*_ isobar at 1 bar.^15,19^ *Periodic boundary conditions correction.^28^

**Table 4:** Performance of the Water Models Using Mean Absolute Percentage Errors MAPE (%) at Different Thermodynamic Conditions^a^

Property	ECCw2024	TIP4P/2005	OPC4	TIP4P-FB
ρ_1bar_	0.072	0.118	0.233	0.085
ρ_300K_	0.173	0.075	0.034	0.012
*D*_OW_	7.0	3.1	7.3	6.6
rdf_1*p*_	1.429	1.429	0.714	1.486
rdf_1*h*_	25.6	24.2	21.5	25.8
η	0.106	2.456	5.982	3.420
γ	5.26	2.17	2.71	3.79
*T*_*melt*_	6.23	8.48	10.3	11.0
*C*_*G*_	0.262	0.208	0.248	0.260

a Results are provided as mean absolute percentage errors MAPE (%) at different thermodynamic conditions. Radial distribution function (rdf), viscosity (η), and surface tension (γ) correspond to 300 K and 1 bar. The melting point temperature is at 1 bar. Finally, the comparison between water models is done employing the cost function *C*_*G*_, see eq 2, without including the relative permittivity. Lower values of *C*_*G*_ mean better performance of the water model (for comparison, the three-site TIP3P model yields a very high value of *C*_*G*_ = 1.973). Note that the properties used in *C*_*G*_ are provided in Table 2 and that the MAPE values are normalized.

Going into further detail, the oxygen–oxygen radial distribution functions (RDF) are presented in Figure 3A. The four water models yield very similar results, particularly within the first coordination shell. They all fit well the position of the experimental position of the first peak (0.280 nm) but overshoot its height as expected due to the lack of many-body interactions and potentially other effects.

Figure 3B shows the temperature dependence of the density at 1 bar. All models perform well between 300 and 340 K. In addition, our model matches the experiment within ∼2 kg/m^3^ all the way to 240 K. At low temperatures, this fixes the ∼5 kg/m^3^ deviations of TIP4P/2005 and TIP4P-FB (which is already substantially smaller than the deviation of OPC4 that reaches ∼13 kg/m^3^).

The temperature dependence of the water self-diffusion coefficient (*D*_*OW*_) is presented in Figure 3C. At temperatures below ∼320 K, all water models, including the present one, converge to values matching experiments, except for OPC4 that diffuses slightly faster than the other water models. At high temperatures (*T* > 320 K), all water models deviate from experiment in a similar way yielding a somewhat too slow dynamics. Over the whole investigated temperature range, all the models show a very similar performance with a MAPE of ∼7%, see Table 4, except for TIP4P/2005 with a bit smaller MAPE of 3.1%.

With good agreement with experiments that our model has been optimized against, the next question to address is whether the model also predicts correctly other physical properties. To this end, we have computed a set of additional properties, namely ε_*N*_, ρ_300K_, γ, η, and *T*_*melt*_ (see Table 4, Table S2, Figure 3, and Figure S4).

One property our model was not a priori optimized against is the temperature dependence of ε_*N*_, i.e., the nuclear contribution to ε_*r*_, see Figure 3D. Within the present nonpolarizable simulations, it represents the only contribution to the dielectric constant, while as a reference it can be computed by dividing the experimental total dielectric constant ε_*r*_ at a given temperature^15,34^ by the infinite frequency dielectric constant at the same temperatures.^19^ The very good agreement of the present model with experiments in the whole temperature range is remarkable, particularly in comparison to the other water models (Figure 3D).

We also calculated the pressure dependence of the density at 300 K (ρ_300K_), see Figure S4. The response to pressure of our models is slightly offset with respect to the other reference models remaining, however, within 4 kg/m^3^ from experiments in the whole investigated pressure range. Together with the proper description of water densities at different temperatures, this agreement shall result in a correct description of the isobaric (κ_*p*_) and isothermal (α_*T*_) compressibilities. All water models yield very similar values of surface tension within 4 mN/m below the experimental value of 71.68 mN/m at 300 K (Table S2). All considered models also do a good job reproducing the viscosity of water at 300 K and 1 bar, falling slightly short of the experimental value 0.85 mPa·s with values between 0.80 and 0.88 mPa·s (Table S2). Finally, all water models somewhat underestimate the melting point of the *I*_*h*_ ice. Although the present model performs the best (see Figure S3), its melting point still lies 17 K below the experimental value of 273.15 K. Note also that for the TIP4P/2005, TIP4P-FB, and OPC4 water models the reported melting points are consistent (within 3 K) with previously computed values.^35^

Overall, using the presently developed optimization framework that takes advantage of AI machinery, we were able to sample efficiently the water parameter phase space and produce a four-site water model compatible with the ECC framework (i.e., possessing ε_*r*_ ≈ 45) with a very good performance, which is comparable with that of currently widely employed four-site water models such as TIP4P/2005, OPC4, or TIP4P-FB. It should be stressed that it was by no means obvious from the onset that it is at all possible to generate a nonpolarizable water model with such a low value of a dielectric constant (truly reflecting only the contribution from nuclear motions) that reproduces experimental properties of liquid water so well. Most importantly, we identified a sizable region of the parameter space encompassing this model that yields high-quality ECC-compatible water models. This will allow us to perform future modifications of the water model if needed to accommodate solutes within the charge scaling ECC approach, such as simple ions or charged biomolecules (or fragments thereof).

## Supporting Information Available

Detailed information about calculations of properties such as the melting point and the dielectric constant ε_*N*_ as well as the absolute optimization results for the water models employed (PDF)
Transparent Peer Review report available (PDF)
