# Supplementary material for: Building Water Models Compatible with Charge Scaling Molecular Dynamics
Source: J Phys Chem Lett. 2024 Mar 7;15(10):2922–8. doi: 10.1021/acs.jpclett.4c00344 (PMC10945568; doi:10.1021/acs.jpclett.4c00344)
Supplement: Supplementary file 1 — jz4c00344_si_001.pdf [file jz4c00344_si_001.pdf]

Supporting Information for: Building Water Models Compatible with Charge Scaling Molecular Dynamics

Victor Cruces Chamorro, Pavel Jungwirth,* and Hector Martinez-Seara*

Institute of Organic Chemistry and Biochemistry, Czech Academy of Sciences, Flemingovo nám. 2, 16610 Prague 6, Czech Republic

E-mail: pavel.jungwirth@uochb.cas.cz; hseara@gmail.com

Molecular dynamics simulation parameters

All simulations were performed using the GROMACS2019 molecular dynamics package[S1] with 832 water molecules in the cubic simulation box. We employed an interaction cutoff of 1.2 nm for the Particle Mesh Ewals (PME)[S2] and the PME Lennard-Jones schemes that take into account the long-range electrostatic and van der Waals interactions. We used the leapfrog algorithm with a timestep of 2.0 fs and a total simulation time of 21 ns. The first nanosecond was considered equilibration and skipped for the analysis.

The simulations were carried out with the isothermic-isobaric (NpT) ensemble, using the Nosé-Hoover thermostat[S3] and the Parrinello-Rahman barostat.[S4] The relaxation times were of 1.0 ps and 5.0 ps, respectively. The compressibility value used with the barostat was 5E-5 bar−1.

Additional information optimization framework

To refine the search for good water models in the already sampled bounded region of the parameters space, we extended our computational framework to extensively use the prediction capabilities of the machine learning model (ML) created during the previous phases. In this refinement phase, we performed 40 independent runs. Each run started from 40 to 100 randomly generated parameter sets inside the set boundaries (named as DE group), and their cost function was evaluated using the ML algorithm. Those groups were used to feed the genetic algorithm, which created between 20 and 40 parameter sets via permutations. According to the neural network, the most suitable parameter set for each DE group was used to substitute the parent or previous point to feed the differential algorithm. This procedure was repeated until the differential evolution converged or the maximum iteration (5000) was reached. The best 2 to 4 models, depending on the predicted cost function value by the ML algorithm, for each group were further simulated using Molecular Dynamics. Figure S1 shows the scheme of this refinement method.

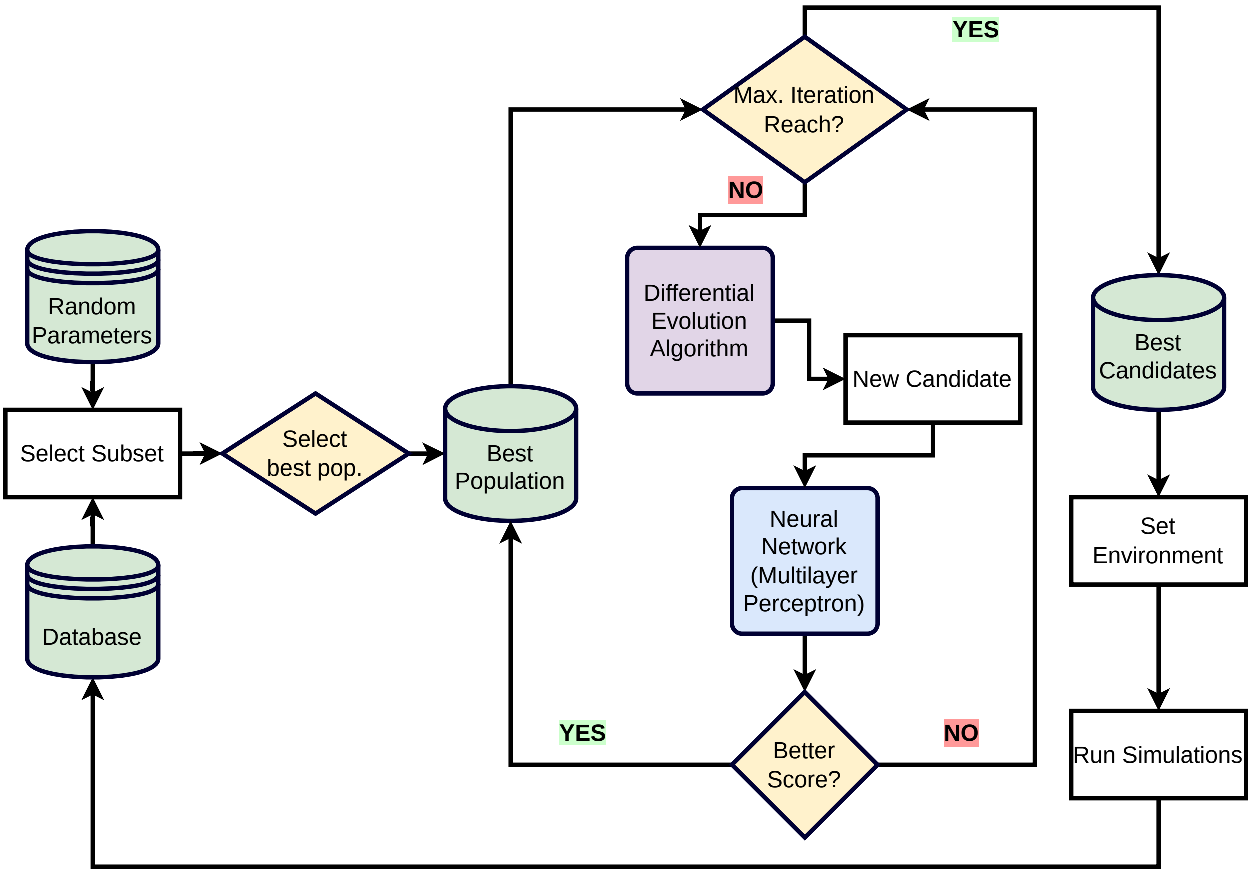

Figure S1: Refinement method Scheme

The models obtained improved substantially from the ones obtained in the previous round with a fraction of the cost as used only less than 150 additional simulations. Of course, the results depend on the previous sampling of the parameters phase space as it determines the quality of the neural network used. For that reason, this refinement was employed once the ML was properly trained with a sufficient population to avoid possible overfitting or fake positive parameters.

The sampling ranges and efficiency of our whole framework is shown in Figure S2.

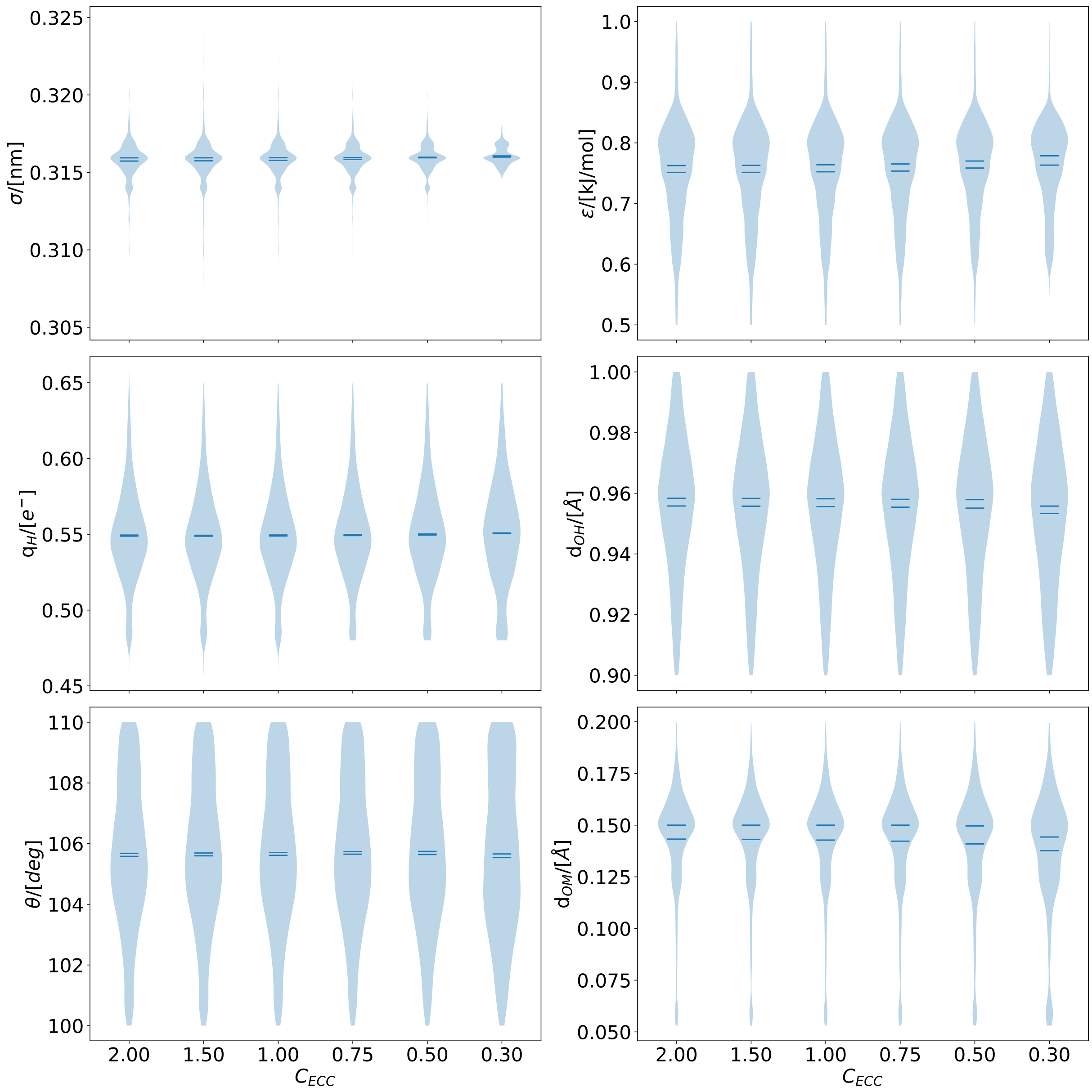

Figure S2: Violinplots of the sampled ECC compatible water model parameters as a function of their maximum cost value, C_{ECC}.

Extended analysis methods

We have calculated some properties at different thermodynamic conditions, which are in Table S2.

The nuclear contribution (ϵ_N) of the dielectric constant at 1 bar was calculated by removing the electronic contribution (ϵ_e) to the relative permittivity (ϵ_r).[S5,S6]

$$\epsilon_N(T) \approx \frac{\epsilon_r(T)}{\epsilon_e(T)} \tag{S1}$$

where ϵ_e is approximated as the refractive index of the medium for the sodium D-line at 589 nm ($\epsilon_e \approx n^2$), and the relative permittivity was calculated using the fluctuation of the total dipole moment of the simulation box,[S7] see equation S2.

$$\epsilon_r = 1 + \frac{4\pi}{3Vk_bT}(\langle M^2\rangle - \langle M\rangle^2) \tag{S2}$$

The reference experimental values are taken from Table S1.

Table S1: Experiment values for relative permittivity ϵ_r[S5,S8] and refractive index.[S9] Those properties are required to calculate the nuclear contribution of the relative permittivity ϵ_N, see equation S1.

Temperature/[K]	ϵ_r	n
270	89.01	1.33430
280	85.04	1.33420
290	81.26	1.33363
300	77.65	1.33267
310	74.20	1.33141
320	70.91	1.32989
330	67.77	1.32815
340	64.77	1.32623
350	61.90	1.32413
360	59.15	1.32185
370	56.53	1.31936

Extended results of the ECCw2024 water models and its comparison with the reference water models

We have calculated the melting point of the water models in the main text by the Direct Coexistence Method (DCM). We have employed the methodology in reference Conde et al.[S10]. The direct coexistence results are in Figure S3.

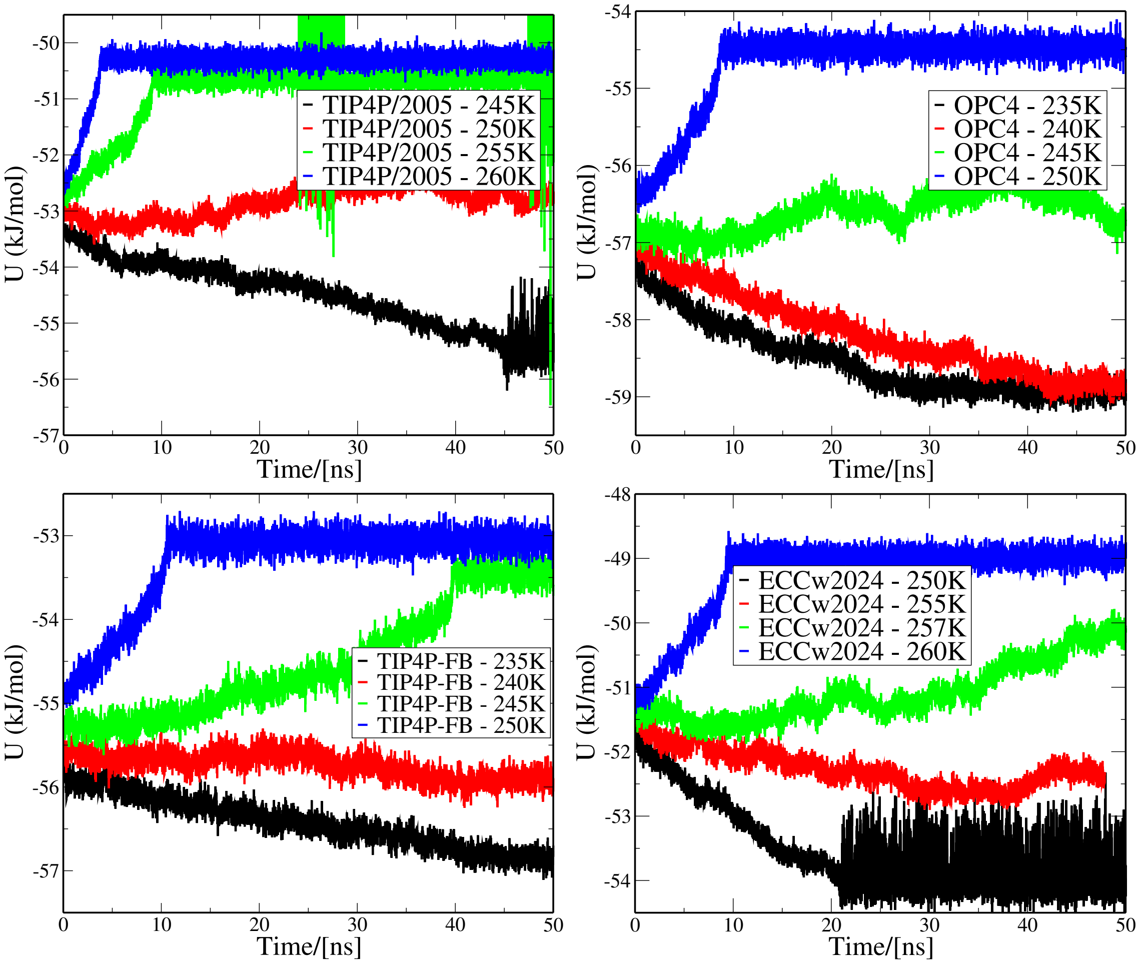

Figure S3: Potential Energy of all water models. A decrease in the potential energy means that the ice I_h phase is growing. Meanwhile, an increment means that the liquid phase is growing. The temperatures where the potential energy remains more or less constant are the coexistence point or melting point.

Figure S4 shows the performance of TIP4P/2005, OPC4, TIP4P-FB, and ECCw2024 at the 300 K isotherm.

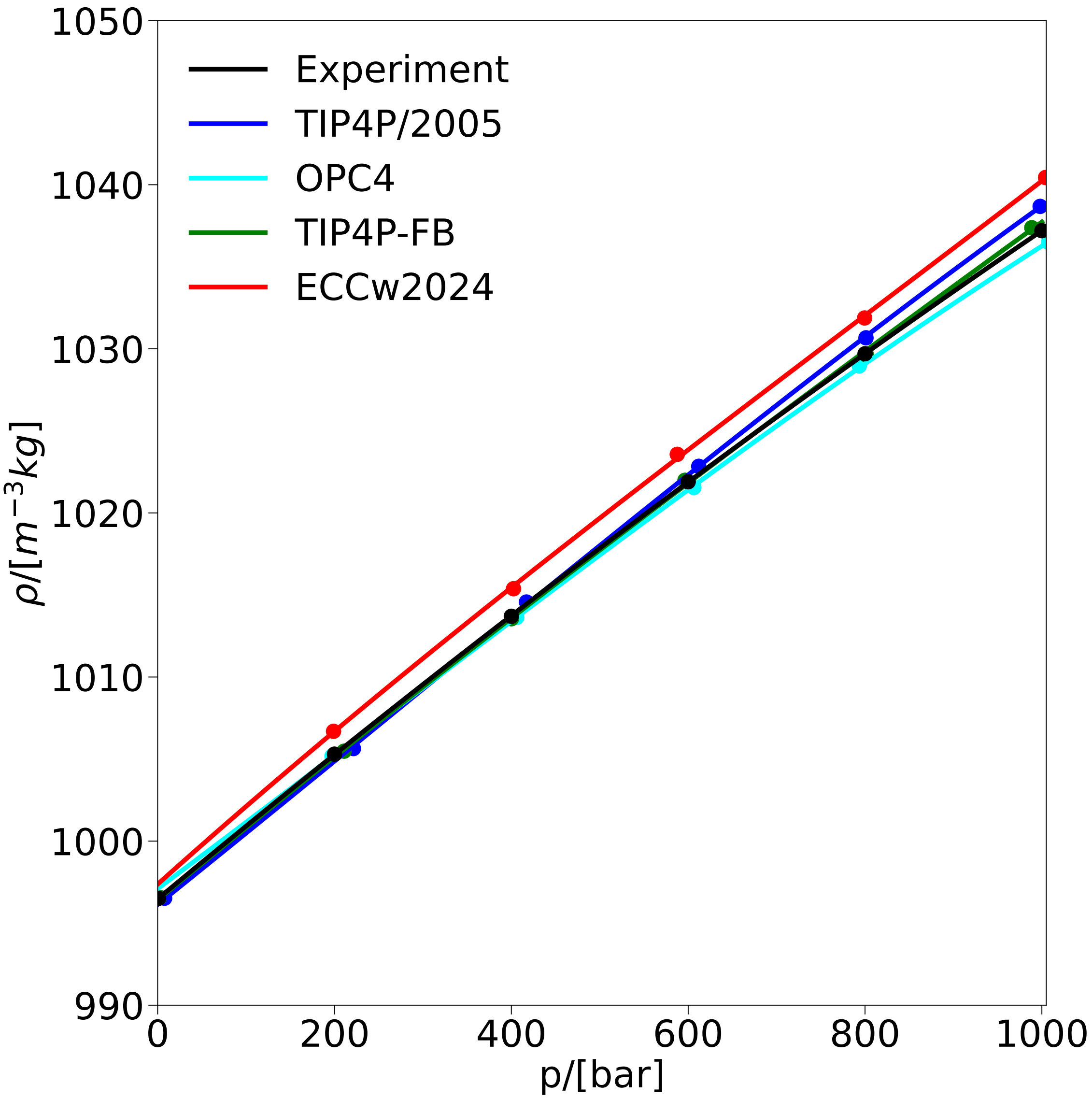

Figure S4: Density of TIP4P/2005, OPC4, TIP4P-FB, and ECCw2024 (this work) water models and the experimental density at 300K and different pressures.[S11]

Table S2 shows the numerical results of all properties calculated for TIP4P/2005, OPC4, TIP4P-FB, and ECCw2024.

Table S2: Performance results of TIP4P/2005, OPC4, TIP4P-FB, and ECCw2024 in comparison with the experimental value or its polynomial interpolation.

Property	T/[K]	p/[bar]	Expt.	TIP4P/2005	OPC4	TIP4P-FB	ECCw2024
$\rho/[kg/m^3]$	240	1.0	980.9	985.7	993.3	985.2	982.4
$\rho/[kg/m^3]$	250	1.0	990.5	993.0	998.6	992.4	991.3
$\rho/[kg/m^3]$	260	1.0	996.5	997.9	1001.0	996.8	996.8
$\rho/kg/m^3]$	270	1.0	999.4	999.7	1001.6	999.1	999.6
$\rho/[kg/m^3]$	280	1.0	1000.2	1000.3	1001.1	999.4	1000.4
$\rho/[kg/m^3]$	290	1.0	999.1	998.9	999.1	998.7	999.3
$\rho/[kg/m^3]$	300	1.0	996.7	996.5	996.6	996.6	997.0
$\rho/[kg/m^3]$	310	1.0	993.4	993.1	993.3	993.3	993.8
$\rho/[kg/m^3]$	320	1.0	989.3	989.1	989.4	990.0	989.6
$\rho/[kg/m^3]$	330	1.0	984.6	984.4	984.8	985.4	985.0
$\rho/[kg/m^3]$	340	1.0	979.5	978.4	979.8	979.9	978.9
$\rho/[kg/m^3]$	350	1.0	973.9	972.7	974.4	974.3	972.8
$\rho/[kg/m^3]$	360	1.0	967.6	965.8	968.4	967.4	966.0
$\rho/[kg/m^3]$	370	1.0	960.5	958.6	962.3	961.4	958.5
$\rho/[kg/m^3]$	300	200	1005.3	1005.6	1005.2	1005.5	1006.7
$\rho/[kg/m^3]$	300	400	1013.7	1014.6	1013.6	1013.6	1015.4
$\rho/[kg/m^3]$	300	600	1021.9	1022.8	1021.6	1022.0	1023.6
$\rho/[kg/m^3]$	300	800	1029.7	1030.7	1029.0	1029.6	1031.9
$\rho/[kg/m^3]$	300	1000	1037.2	1038.7	1036.5	1037.4	1040.4
$D_{OW}[cm^2/s]$	270	1.0	9.80E-6	9.01E-6	1.04E-5	8.50E-5	8.23E-6
$D_{OW}[cm^2/s]$	280	1.0	1.38E-5	1.25E-5	1.37E-5	1.22E-5	1.16E-5
$D_{OW}[cm^2/s]$	290	1.0	1.86E-5	1.68E-5	1.78E-5	1.62E-5	1.55E-5
$D_{OW}[cm^2/s]$	300	1.0	2.41E-5	2.13E-5	2.24E-5	2.07E-5	2.02E-5
$D_{OW}[cm^2/s]$	310	1.0	3.03E-5	2.67E-5	2.72E-5	2.53E-5	2.49E-5
$D_{OW}[cm^2/s]$	320	1.0	3.72E-5	3.25E-5	3.24E-5	3.11E-5	3.13E-5
$D_{OW}[cm^2/s]$	330	1.0	4.49E-5	3.89E-5	3.87E-5	3.73E-5	3.86E-5
$D_{OW}[cm^2/s]$	340	1.0	5.33E-5	4.61E-5	4.48E-5	4.31E-5	4.43E-5
$D_{OW}[cm^2/s]$	350	1.0	6.25E-5	5.27E-5	5.12E-5	5.03E-5	5.14E-5
$D_{OW}[cm^2/s]$	360	1.0	7.25E-5	6.12E-5	5.87E-5	4.84E-5	5.90E-5
$D_{OW}[cm^2/s]$	370	1.0	8.21E-5	6.77E-5	6.70E-5	6.68E-5	6.87E-5
rdf_{1p}[Å]	300	1.0	2.80	2.78	2.80	2.78	2.76
rdf_{1h}[a.u]	300	1.0	2.58	3.21	3.19	3.23	3.24
$\eta[mPa \cdot s]$	300	1.0	0.8509	0.83	0.80	0.88	0.85
$\gamma[mN/m]$	300	1.0	71.68	70.1	69.7	69.0	68.3
ϵ_r	300	1.0	78.4	58.2	77.4	77.3	44.3
$T_{melt}/[K]$	—	1.0	273.15	250	245	243	256
$TMD/[K]$	—	1.0	277	277	270	279	279

References

[S1] Abraham, M. J.; Murtola, T.; Schulz, R.; Páll, S.; Smith, J. C.; Hess, B.; Lindahl, E. GROMACS: High performance molecular simulations through multi-level parallelism from laptops to supercomputers. *SoftwareX* **2015**, *1-2*, 19–25.

[S2] Essmann, U.; Perera, L.; Berkowitz, M. L.; Darden, T.; Lee, H.; Pedersen, L. G. A Smooth Particle Mesh Ewald Method. *J. Chem. Phys.* **1995**, *103*, 8577–8593.

[S3] Hoover, W. G. Canonical Dynamics: Equilibrium Phase-Space Distributions. *Phys. Rev. A* **1985**, *31*, 1695–1697.

[S4] Parrinello, M.; Rahman, A. Polymorphic transitions in single crystals: A new molecular dynamics method. *J. Appl. Phys.* **1981**, *52*, 7182–7190.

[S5] Fernández, D. P.; Goodwin, A. R. H.; Lemmon, E. W.; Levelt Sengers, J. M. H.; Williams, R. C. A Formulation for the Static Permittivity of Water and Steam at Temperatures from 238 K to 873 K at Pressures up to 1200 MPa, Including Derivatives and Debye–Hückel Coefficients. *J. Phys. Chem. Ref. Data* **1997**, *26*, 1125–1166.

[S6] Leontyev, I. V.; Stuchebrukhov, A. A. Electronic continuum model for molecular dynamics simulations. *J. Chem. Phys.* **2009**, *130*, 085102.

[S7] Neumann, M. Dipole Moment Fluctuation Formulas in Computer Simulations of Polar Systems. *Mol. Phys.* **1983**, *50*, 841–858.

[S8] Malmberg, C. G.; Maryott, A. A. Dielectric Constant of Water from 0 to 100 C. *J. Res. Natl. Bur. Stand.* **1956**, *56*, 1.

[S9] Schiebener, P.; Straub, J.; Levelt Sengers, J. M. H.; Gallagher, J. S. Refractive Index of Water and Steam As Function of Wavelength, Temperature and Density. *J. Phys. Chem. Ref. Data* **1990**, *19*, 677–717.

[S10] Conde, M. M.; Gonzalez, M. A.; Abascal, J. L. F.; Vega, C. Determining the phase diagram of water from direct coexistence simulations: The phase diagram of the TIP4P/2005 model revisited. *J. Chem. Phys.* **2013**, *139*, 154505.

[S11] Wagner, W.; Pruß, A. The IAPWS Formulation 1995 for the Thermodynamic Properties of Ordinary Water Substance for General and Scientific Use. *J. Phys. Chem. Ref. Data* **2002**, *31*, 387–535.
