# Supplementary material for: Building Water Models Compatible with Charge Scaling Molecular Dynamics
Source: J Phys Chem Lett. 2024 Mar 7;15(10):2922–8. doi: 10.1021/acs.jpclett.4c00344 (PMC10945568; doi:10.1021/acs.jpclett.4c00344)
Supplement: Supplementary file 2 — jz4c00344_si_002.pdf [file jz4c00344_si_002.pdf]

jz-2024-00344h.R1

Name: Peer Review Information for "Building Water Models Compatible With Charge Scaling Molecular Dynamics"

First Round of Reviewer Comments

Reviewer: 1

Comments to the Author

The problem of all existing nonpolarizable water models with their value of permittivity has been recently well discussed in the literature and the call for a model consistent with ECC correction effectively implementing the electronic polarizability became a must. While definitely some groups will continue to optimize ionic potentials with existing (intrinsically internally inconsistent) water models, the submitted manuscript paves the way to go – or at least presents for the first time excellent solid results following the previously outlined path.

I understand that the authors deserve to claim the applied optimization approach and share the great news that a consistent water model can be found and performs well, in most aspects superior to the other successful water models. I'm also glad that the authors implicitly on numerous places and explicitly in the final paragraph admit, that even better model (or several of them optimized for different properties/conditions) might result from continuation of this work, particularly when taking into account interactions in multicomponent systems.

The scientific relevance of this manuscript is enormous and I evaluate the scientific quality of the manuscript (the key computing part) as excellent.

In strong contrast is the huge number of minor scientific and typographic errors. The authors should carefully read the manuscript and correct not only those deficiencies that I identify below, but also others, which I do not list or spot.

This work should be published after a revision correcting numerous negligence errors/inconsistencies in the manuscript and I hope that the authors will also consider some of my scientific recommendations.

Scientific points:

1) I find misleading the sentence "First simulations involving water data back to the early days of FFMD.[2]" followed by a reference to a 2003 paper. A reference to much older paper, mentioning the era (70's ?) or making clear that the 2003 paper summarizes the decades of development, would be more appropriate.

2) References to the machine learning and differential evolution algorithms applied, and the ReLu activation function should be added to the manuscript or SI.

3) I strongly recommend to label the complete scoring function, eq. (2), as C (or otherwise, e.g. E for error), but not $C_{□r}$ (eq. (2)) or $C_{□}$ (Caption of Figure S2). For some time I was afraid when analyzing Fig. 2 and reading the

r

text in the RHS of page 4, that only the error of predicted $□_r$ is discussed, then thought that $C_{□r}$ should be C_{er} or C_{err} to indicate the error, before realizing that it is the overall weighted error including evaluation of $□_r$. Confusing enough – moreover the authors refer to this property inconsistently on p. 4 LHS, l. 18 – "i.e., the global score (C) is calculated using a weighted average, see equation 2".

Subsequently, the authors would need to change labeling of the cost function without the $□_r$ term in Table 4. It is not clear if the cost function C reported in Table 4 is obtained just by eliminating the $□_r$ term (i.e. the weights do not sum to 1or by proportionally reweighting the terms.

4) The authors should stick to the notation introduced in eq. (1) and apply it throughout the manuscript. Therefore, $□_r$ in Table 2; p. 4 RHS, l. 3; p. 4 RHS l. 41 (optional), and eq. (S1) should be $□_N$ – true not only for ECCw2024, but also for other models (where $□_r$ =$□_N$ if ECC is not applied, or $□_r$ =$□_N$ $□_e$, where $□_e$ depends on the ECC scaling factor rather than refractive index. To bring more confusion, the authors use notation $□_N$, $□_{nuc}$ (p. 6 LHS, l. 47, l. 50), and $□_{Nuc}$ (eq. (S2)). BTW I would prefer $□_n$ when the electronic part is denoted as $□_e$ (or $□_{\mathrm{nuc}}$ and $□_{\mathrm{ele}}$). These inconsistencies combined bewilder the reader in one of the main ideas of this manuscript – but the correction is easy.

5) When discussing the "configurations" in this manuscript, the author should better elucidate that by configuration they mean parametrization (i.e. the set of 6 parameter values, configuration in a parametric space), as opposed to molecular configuration (molecular structure).

6) On p. 5, l. 53 the authors comment the OW-OW RDF: "They all fit well the position of the experimental position of the first peak (0.280 nm) but overshoot its height as expected due to the lack of many-body interactions." Knowing this, would not it be better not to include this height as one of the fitting parameters and rather replace it by coordination number up to the first minimum or some other quantity with more direct impact on the performance of the model? In many cases the height, resp. width is not critical, but the integral is.

7) In the manuscript or SI, it should be stressed, that the atomic charges (and consequently the dipole and quadrupole moments) reported in the manuscript are not the 'true' molecular properties, but effective ones, resulting from the common practice in ECC simulations that electronic permittivity $□_{\mathrm{e}}$ is applied only to reducing the interactions among ions, but reduced effective charges and no reduction of the interactions by $□_{\mathrm{e}}$ are applied to neutral molecules including water – see e.g. https://doi.org/10.1080/00268976.2015.1005191, https://doi.org/10.1016/j.molliq.2020.113571.

As a result (ECCw2024 makes things simple due to being fully ECC-compliant), the 'real' charge q_{H} is not

0.606 e as reported in Table 3, but 0.606 × $(1.78)^{0.5}$ = 0.809 e and the 'real' dipole moment is not 2.168 D, but

2.168 × (1.78)$^{0.5}$ =2.89 D, which in fact perfectly agrees with the experimental value 2.9 ± 0.6 D [https://doi.org/10.1063/1.481541].

8) References to some experimental data in Figure 3 are missing. I dislike the label 'Exp. Hummer-Yeh', since Hummer and Yeh did not carry out an experiment. I suggest 'Exp. ref. value' and comment the usage of correction for finite box size in the caption.
9) When absolute values of properties are discussed (p.6 RHS, l. 41, 45), the reader should not be referred to Table 4 (where only relative deviations are provided), but to Table S1.
10) The energies in Figure S3 are not in kJ/mol, but multiplied by the number of molecules (as Gromacs does). I recommend getting rid of this multiplication. The energies still seem to me too negative if 832 molecules were applied…

Other points (both scientific and typographic):

11) I suggest improving the TOC Graphic (particularly the □$_r$ ~45 label spanning the box is ugly).
12) TIP4P-2005 (the abstract and SI) should be TIP4P/2005.
13) "oxygen–dummy atom d_{OH} distances" should be "oxygen–dummy atom d_{OM} distances" – page 2, line 2, right column.
14) The notation of *Angle* for the □HOH angle is awkward. Either use □HOH or give it a name (standardly □). 15) Units of angle should be given as "deg" – not "Deg" or "Degree" (e.g. Table 1, Fig. 2, …)
16) Units should be given in non-italics, quantities in italics and their indices which do not denote mathematical symbols but text in non-italics (e.g. d_{OH}). These basic rules are inconsistently violated throughout the manuscript and SI. See e.g. Table 1. Table 2, p. 4 RHS …
17) The authors have a problem with the fact that charges in molecular simulations are given in units of elementary charge [e]. See p. 4 RHS, l. 16; Table 1, Table 3, Figure 2.
18) The common non-SI unit of a dipole moment is standardly denoted as D, not De (Table 3).
19) The property denoted as Q_T in Table 3 is not explained (presumably the quadrupole moment in the bisector directions). Also, it would make sense to separate (by a line) the 6 input parameters from the last two quantities, which are derived from them (and are not independent parameters in the context of model optimization).
20) The labels in figures and Table 4 are given in strange notation, e.g. T/[K] (see Figure 3, Figure 2, Figure S4, Table S1 – cf. Figure S3), which I find highly nonstandard and awkward. Either T [K] or T/K, not the hybrid.
21) The units of densities and diffusivities are missing in Table 4, unit of rdf_{1h} is not [a.u.], but dimensionless (also Table S1). Since the reported values are relative errors (and temperature in K), the units are in fact unimportant – but clearly there is a mismatch in this table.
22) Strange section title "Additional information optimization framework" – p. 2 SI.
23) The word 'Meanwhile' in the Caption of Figure S3 should be rather 'Conversely', 'Vice versa', etc.
24) The unit of diffusivity "$cm^{-2}s$" in Table S1 is wrong. Should be "cm^2/s" or even better "$10^{-9}m^2/s$" or "nm^2/ns".

Reviewer: 2

Comments to the Author

This is a straightfoward yet important study on the optimization of water force field. Using simple machine learning techniques the authors were able to generate a four-point water model that outperforms many existing ones. They also showed potential of its general application in other aqueous systems as a relatively wide range of parameters were identified to reproduce reasonably physical properties of water. I have only two minor comments/questions:

1. The caption of Table 4 "All properties are at 300 K and 1 bar expcept..." is a little misleading when one examimes the data on Tmelt. The authors might want to rephrase.

2. Is there a more qualitative criterion to judge how well a sampling covers the parameter space? A more detailed discussion will help readers understand the merit of the optimization process.

Reviewer: 3

Comments to the Author

In this paper a new water model is proposed (rigid and non-polarizable) that

performs as well as the best available so far (OPC, tip4p/2005 and tip4p-fb) but

with a dielectric constant of 45 for water (compared to the experimental value

of 78). Although not reproducing the experimental value of the dielectric constant

of water seems estrange at first, this is not so when one considers that non-polarizable models are missing the electronic contribution to the dielectric constant

as given by the high frequency value of the dielectric constant which is related to the refraction index.

Overall the model is quite good and the author show that it was possible

to reproduce the properties of water with models having a dielectric constant

between 45 and 78. In the future the authors will propose a force field based on

this ECC approach.

The calculations were done carefully and they are robust. Besides results

are given in tabular form in the Supplementary Material. Overall I recommend the

publication of this interesting work. Nice work !

However, I have some comments that need to be addressed before recommending the final acceptance of the paper :

1) The idea of having a water model that is not reproducing the dielectric constant of water may find certain resistance in our community. I think the authors could state, that in addition to the ECC formulation,another way of arriving to similar ideas is to state that PES and DMS are different and typically the true dipole moment of water in condensed matter is higher than this used by common water models (Molecular Physics 113 1145 (2015)). Maybe the authors would like to comment that briefly in the introduction.

2) I do not understand Table 4, which is one of the most important results of the paper.

a) First of all it would be useful to state in the caption that low values of C and

of any properties means good performance of the model (i.e good agreement with experiment).

b) In Eq.(2) of the main text five properties are used to determine C. I have the feeling though that value of C of the first line in Table 4 is using the 8 properties provided in Table 4 (with the same weight to all properties ? with different weights ?) . Clarify. Please provide the formula in the main text that allows the reader to obtain by himself/herself the value of C reported in the first line of Table 4.

c) For each property a certain score is provided (for instance, for D_OW 7, 3.3, 7.5, 7.0)

How are these numbers obtained ? Please provide also the recipe that allows the reader to understand how the numbers of each line of Table 4 were obtained.

d) Besides I believe there are inconsistencies between the score of Table 4 and the results of Supplementary Material.

Let us take viscosity as an example (although I think it happens also for other properties as rdf_1p). Please check this.

Table 4

Property	ECCw20024	tip4p/2005	opc4	tip4p-fb
Viscosity	0.106	2.456	3.420	0.106

Supplementary material

	Exp	tip4p/2005	opc4	tip4p-fb	ECCw2024
Viscosity	0.8509	0.83	0.80	0.88	0.85

How is it possible that ECCw2024 and tip4p-fb have the same score for the viscosity (0.106) if the viscosity of ECCw2024 is 0.85 , tip4p-fb 0.88 and the experimental value is 0.8509 ?

ECCw2024 is very close to the experimental value (0.85) but tip4p-fb (0.88) is not. Besides tip4p/2005 is closer to experiment than tip4p-fb and it obtains a worse score.

3) State clearly in the caption of Table 4 and in the supplementary material that all results were obtained with a cutoff of 12 A and using PME for both the electrostatics and for the LJ part of the potential.

Also it should be stated clearly in the supplementary pdf file, how these results were obtained (i.e. number of particles ,

cutoff, and whether PME was used for both the electrostatics and for the LJ part of the potential). This is mentioned in

the main text but it should be stated again in the supplementary material as the supplementary material can be obtained as

a separate pdf file and I think it is important to state in this file how the results were obtained.

4) This is optional, but in the literature the model labeled as OPC4 in this work is denoted as OPC in the literature. Is it worth to change the notation ? That could be confusing for certain readers.

5) Please provide in Tabular form the experimental values at infinite frequency (epsilon_el(T)) used in Equation

S2 of the supplementary material.

6) This is not the first work where the melting point of ice Ih for the tip4p-fb was obtained. It was determined previously (J.Chem.Phys. 156 216101 (2022)).

In fact the value reported here 243K is consistent with that published previously, 242K. That should be mentioned. Also the melting temperature obtained here for OPC (245K) seems to be 3K larger than that reported by Onufriev et al. (242.2K) . That should be commented.

7) I think it would be work to have in parenthesis in the Supplementary material the values of the diffusion coefficients obtained for the models considered in this work after including the Yeh Hummer correction (after all viscosities

of all the models are reasonable so that one could use the experimental value of the viscosity for the YH correction).

I am not against having the results for D for the size considered in this work, but including the value that would be obtained using Yeh and Hummer corrections (for instance in parenthesis) would be useful.

8) Probably nuclear quantum effects are also responsible of the overestimate of the first peak of the g(r) and not only many body effects. I do not agree about assigning the high peak of g(r) only to many body forces.

9) Could the authors report in the Supplementary File the exact location of the TMD (temperature of the maximum in density) as obtained in this work ?

10) In Table S1, in the last line the melting point is reported for different water models. Please remove 300K in the second column

(having 1 bar in the third column is fine). Obviously the authors are reporting the melting temperature, and 300K should be removed.

Author's Response to Peer Review Comments:

Dear Editor,

Thank you very much for your e-mail and referee reports concerning our ms. "Building Water Models Compatible With Charge Scaling Molecular Dynamics". We are pleased by the positive views of the reviewers concerning our work and, at the same, time took into consideration all the points that they raised (as detailed below). We also followed all the editorial comments and corrected the ms. accordingly.

With best regards
pavel

Ad Reviewer 1: We are grateful to the reviewer for appreciating the scientific quiality of our work. At the same time we addressed all the points raised as detailed below:
Reviewer comments (in blue):
1) I find misleading the sentence “First simulations involving water data back to the early days of FFMD.2” followed by a reference to a 2003 paper. A reference to much older paper, mentioning the era (70’s ?) or making clear that the 2003 paper summarizes the decades of development, would be more appropriate.
Authors’ reply (in bold, with new text added to the ms. highlighted in red):
We added references to the original papers on early water models.
2) References to the machine learning and differential evolution algorithms applied, and the ReLu activation function should be added to the manuscript or SI.
Authors’ reply:
We added references to the ML and DE algorithms.
3) I strongly recommend to label the complete scoring function, eq. (2), as *C* (or otherwise, e.g. *E* for error), but not *C*□*r* (eq. (2)) or *C*□*r* (Caption of Figure S2). For some time I was afraid when analyzing Fig. 2 and reading the text in the RHS of page 4, that only the error of predicted □*r* is discussed, then thought that *C*□*r* should be *C*er or *C*err to indicate the error, before realizing that it is the overall weighted error including evaluation of □*r*. Confusing enough – moreover the authors refer to this property inconsistently on p. 4 LHS, l. 18 – “i.e., the global score (C) is calculated using a weighted average, see equation 2”.
Subsequently, the authors would need to change labeling of the cost function without the □*r* term in Table 4. It is not clear if the cost function *C* reported in Table 4 is obtained just by eliminating the □*r* term (i.e. the weights do not sum to 1or by proportionally reweighting the terms.
Authors’ reply:
We unified the labeling of the scoring function.
4) The authors should stick to the notation introduced in eq. (1) and apply it throughout the manuscript. Therefore, □*r* in Table 2; p. 4 RHS, l. 3; p. 4 RHS l. 41 (optional), and eq. (S1) should be □*N* – true not only for ECCw2024, but also for other models (where □*r* =□*N* if ECC is not applied, or □*r* =□*N* □*e*, where □*e* depends on the ECC scaling factor rather than refractive index. To bring more confusion, the authors use notation □*N*, □*nuc* (p. 6 LHS, l. 47, l. 50), and □*Nuc* (eq. (S2)). BTW I would prefer □*n* when the electronic part is denoted as □*e* (or □nuc

and □ele). These inconsistencies combined bewilder the reader in one of the main ideas of this manuscript – but the correction is easy.
Authors' reply:
We consistently unified the labeling of the nuclear part of the dielectric constant.

5) When discussing the "configurations" in this manuscript, the author should better elucidate that by configuration they mean parametrization (i.e. the set of 6 parameter values, configuration in a parametric space), as opposed to molecular configuration (molecular structure).
Authors' reply:
To avoid confusion, we now consistently use the term points in the parameter space.

6) On p. 5, l. 53 the authors comment the OW-OW RDF: "They all fit well the position of the experimental position of the first peak (0.280 nm) but overshoot its height as expected due to the lack of many-body interactions." Knowing this, would not it be better not to include this height as one of the fitting parameters and on the performance of the model? In many cases the height, resp. width is not critical, but the integral is.
Authors' reply: The integral over the first peak of the RDF, i.e., the coordination number is unfortunately ill-defined (since it depends on an arbitrarily chosen end point of the integration), therefore, it cannot be reliably used as a fitting parameter. The fact that all the good 4-site models consistently underestimate the height of the first peak of the RDF points to the fact that that this is just a constant offset that does not impact optimization of other properties.

7) In the manuscript or SI, it should be stressed, that the atomic charges (and consequently the dipole and quadrupole moments) reported in the manuscript are not the 'true' molecular properties, but effective ones, resulting from the common practice in ECC simulations that electronic permittivity □e is applied only to reducing the interactions among ions, but reduced effective charges and no reduction of the interactions by □e are applied to neutral molecules including water – see e.g. https://doi.org/10.1080/00268976.2015.1005191, https://doi.org/10.1016/j.molliq.2020.113571. As a result (ECCw2024 makes things simple due to being fully ECC-compliant), the 'real' charge *q*H is not 0.606 e as reported in Table 3, but 0.606 × (1.78)0.5 = 0.809 e and the 'real' dipole moment is not 2.168 D, but 2.168 × (1.78)0.5 =2.89 D, which in fact perfectly agrees with the experimental value 2.9 ± 0.6 D
Authors' reply: We thank the reviewer for pointing out this very positive feature of our model. However, the dipole moment of a water molecule in the liquid bulk is strictly speaking not a measurable quantity, so we do not want to overinterpret this feature.

8) References to some experimental data in Figure 3 are missing. I dislike the label 'Exp. Hummer-Yeh', since Hummer and Yeh did not carry out an experiment. I suggest 'Exp. ref. value' and comment the usage of correction for finite box size in the caption.
Authors' reply: A reference to experimental data was added and the label was modified.

9) When absolute values of properties are discussed (p.6 RHS, l. 41, 45), the reader should not be referred to Table 4 (where only relative deviations are provided), but to Table S1.
Authors' reply: Fixed.
10) The energies in Figure S3 are not in kJ/mol, but multiplied by the number of molecules (as Gromacs does). I recommend getting rid of this multiplication. The energies still seem to me too negative if 832 molecules were applied…
Authors' reply: Fixed.
Minor (mostly typographic) points 11) - 24)
Authors' reply: All fixed.

Ad Reviewer 2: We appreciate reviewer's positive view and addressed the two minor issues raised as detailed below:

Reviewer comments:

1. The caption of Table 4 "All properties are at 300 K and 1 bar expcept..." is a little misleading when one examimes the data on Tmelt. The authors might want to rephrase.
Authors' reply: The critical sentence in the caption of Table 4 has been modified. It now reads "Results are provided as mean absolute percentage errors MAPE (%) at different thermodynamic conditions. Radial distribution function (rdf), viscosity (η), and surface tension (γ) correspond to 300 K and 1 bar.". Additionally, table 4 has been updated improved.
2. Is there a more qualitative criterion to judge how well a sampling covers the parameter space? A more detailed discussion will help readers understand the merit of the optimization process.
Authors' reply: Unfortunately, within the ML approaches this is rather hard to assess quantitatively.
Ad Reviewer 3: We appreciate reviewer's positive view (Nice work!) and at the same time we addressed all the minor issues as detailed below:

Reviewer comments:

1) The idea of having a water model that is not reproducing the dielectric constant of water may find certain resistance in our community. I think the authors could state, that in addition to the ECC formulation,another way of arriving to similar ideas is to state that PES and DMS are different and typically the true dipole moment of water in condensed matter is higher than this used by common
water models (Molecular Physics 113 1145 (2015)). Maybe the authors would like to comment that briefly in the introduction.
Authors' reply: We added to the ms. the above reference together with the following sentence (p. 2, l. 41) "They also possess water dipole moments larger than the gas phase value (albeit typically smaller than the value in the liquid)."
2) I do not understand Table 4, which is one of the most important results of the paper.

a) First of all it would be useful to state in the caption that low values of C and of any properties means good performance of the model (i.e good agreement with experiment).

Authors' reply: We added the following sentence to the caption of Table 4 "Lower values of C mean better performance of the model (for comparison, the 3-site TIP3P model yields a very high value of C_G = 1.973)"

b) In Eq.(2) of the main text five properties are used to determine C. I have the feeling though that value of C of the first line in Table 4 is using the 8 properties provided in Table 4 (with the same weight to all properties ? with different weights ?) . Clarify. Please provide the formula in the main text that allows the reader to obtain by himself/herself the value of C reported in the first line of Table 4.

Authors' reply: We have relabeled the cost functions as C_{ECC} for the cost function used during the optimization and C_G for the cost function used to compare water models.

c) For each property a certain score is provided (for instance, for D_OW 7, 3.3, 7.5, 7.0)

How are these numbers obtained ? Please provide also the recipe that allows the reader to understand how the numbers of each line of Table 4 were obtained.

Authors' reply: The cost function is the practically the same for both optimization and comparing water models from the literature. The only difference is that we have not used the dielectric constant for the cost function when comparing water models.

d) Besides I believe there are inconsistencies between the score of Table 4 and the results of Supplementary Material.

Let us take viscosity as an example (although I think it happens also for other properties as rdf_1p). Please check this.

Table 4

Property	ECCw20024	tip4p/2005	opc4	tip4p-fb
Viscosity	0.106	2.456	3.420	0.106

Supplementary material

	Exp	tip4p/2005	opc4	tip4p-fb	ECCw2024
Viscosity	0.8509	0.83	0.80	0.88	0.85

How is it possible that ECCw2024 and tip4p-fb have the same score for the viscosity (0.106) if the viscosity of ECCw2024 is 0.85 , tip4p-fb 0.88 and the experimental value is 0.8509 ?

ECCw2024 is very close to the experimental value (0.85) but tip4p-fb (0.88) is not. Besides tip4p/2005 is closer to experiment than tip4p-fb and it obtains a worse score.

Authors' reply: We checked carefully the results in Table 4, so the table is now updated with minor errors corrected.

3) State clearly in the caption of Table 4 and in the supplementary material that all results were obtained with a cutoff of 12 A and using PME for both the electrostatics and for the LJ part of the potential.

Also it should be stated clearly in the supplementary pdf file, how these results were obtained (i.e. number of particles ,

cutoff, and whether PME was used for both the electrostatics and for the LJ part of the potential). This is mentioned in

the main text but it should be stated again in the supplementary material as the supplementary material can be obtained as

a separate pdf file and I think it is important to state in this file how the results were obtained.

Authors' reply: All the cutoffs are now clearly defined in the ms. and in the SI.

4) This is optional, but in the literature the model labeled as OPC4 in this work is denoted as OPC in the literature. Is it worth to change the notation ? That could be confusing for certain readers.

Authors' reply: There are two OPC models – a 3-site and a 4-site one. To avoid confusion, we stick here to the notation OPC4 to clearly denote the 4-site model tested in our study.

5) Please provide in Tabular form the experimental values at infinite frequency (epsilon_el(T)) used in Equation S2 of the supplementary material.

Authors' reply: The experimental values of the temperature-dependent dielectric constant are now provided in the SI also in a tabular form.

6) This is not the first work where the melting point of ice Ih for the tip4p-fb was obtained. It was determined previously (J.Chem.Phys. 156 216101 (2022)). In fact the value reported here 243K is consistent with that published previously, 242K. That should be mentioned. Also the melting temperature obtained here for OPC (245K) seems to be 3K larger than that reported by Onufriev et al. (242.2K) . That should be commented.

Authors' reply: We added to the ms. the above references with the following sentence (p. 6, l. 49) "Note also that the reported melting points are consistent (within 3 K) with previously computed values for the TIP4P-FB and OPC4 models."

7) I think it would be work to have in parenthesis in the Supplementary material the values of the diffusion coefficients obtained for the models considered in this work after including the Yeh Hummer correction (after all viscosities of all the models are reasonable so that one could use the experimental value of the viscosity for the YH correction).

I am not against having the results for D for the size considered in this work, but including the value that would be obtained using Yeh and Hummer corrections (for instance in parenthesis) would be useful.

Authors' reply: For the corrected values of the diffusion coefficient one would have to calculate the size/dependent diffusion coefficients which is computationally very demanding. That's precisely why we did it in the way presented in the ms.

8) Probably nuclear quantum effects are also responsible of the overestimate of the first peak of the g(r) and not only many body effects. I do not agree about assigning the high peak of g(r) only to many body forces.

Authors' reply: Nuclear quantum effects for O-O RDF are small, but there may be other effects involved. Accordingly, we added to the ms. the following text (p. 5, l. 54) "....and potentially other effects."

9) Could the authors report in the Supplementary File the exact location of the TMD (temperature of the maximum in density) as obtained in this work ?

Authors' reply: The exact location of TMD for the four water models presented is now provided in a tabular form in the SI.

10) In Table S1, in the last line the melting point is reported for different water models. Please remove 300K in the second column (having 1 bar in the third column is fine). Obviously the authors are reporting the melting temperature, and 300K should be removed.

Authors' reply: Fixed.
